# The subaortic tendon as a mimic of hypertrophic cardiomyopathy

**DOI:** 10.1186/1476-7120-7-31

**Published:** 2009-07-03

**Authors:** James Ker

**Affiliations:** 1 Department of Physiology, University of Pretoria, Pretoria, South Africa, PO Box 24318, Gesina, Pretoria, South Africa 0031

## Abstract

Originally described by Brock and Teare, today hypertrophic cardiomyopathy is clinically defined as left (or right) ventricular hypertrophy without a known cardiac or systemic cause, such as systemic hypertension, Fabry's disease or aortic stenosis.

Also appreciated today is the enormous genotypic and phenotypic heterogeneity of this disease with more than 300 mutations over more than 24 genes, encoding various sarcomeric, mitochondrial and calcium-handling proteins, all as genetic causes for hypertrophic cardiomyopathy.

Phenotypically, the disease can vary from negligible to extreme hypertrophy, affecting either the left and/or right ventricle in an apical, midventricular or subaortic location.

Left ventricular false tendons are thin, fibrous or fibromuscular structures that traverse the left ventricular cavity. Recently, a case report was presented where it was shown that such a false tendon, originating from a subaortic location, was responsible for striking ST-segment elevation on the surface electrocardiogram.

In this case report, a case is presented where such a subaortic tendon led to the classic echocardiographic appearance of hypertrophic cardiomyopathy, thus in the assessment of hypertrophic cardiomyopathy, this entity needs to be excluded in order to prevent a false positive diagnosis of hypertrophic cardiomyopathy.

## Introduction

Hypertrophic cardiomyopathy (HCM) was first described in 2 publications between 1957–1959 by Brock [1-3]. During this period Teare [1,4] also described the entity of asymmetrical septal hypertrophy in 8 autopsy cases.

HCM is the most prevalent genetic cardiovascular disease, as it affects one in 500 individuals and exhibits enormous genotypic and phenotypic heterogeneity [5].

Phenotypically, hypertrophy can vary from negligible to extreme – similarly fibrosis and myocyte disarray can also range from negligible to extreme [5]. This phenotypic variation is the result of the vast array of mutations present in the family of HCM [5].

These mutations can be inherited (familial) or can occur de novo (sporadic) [6].

Currently, more than 300 mutations, which are scattered over more than 24 genes are known as causes for HCM [5]. These involved genes encode various proteins of the sarcomere, mitochondria and the calcium-handling apparatus [5]. Sarcomeric mutations can affect the thick myofilament (beta-myosin heavy chain, regulatory myosin light chain and essential myosin light chain), the intermediate myofilament (myosin binding protein C) or the thin myofilament (cardiac troponins T and I, alpha-tropomyosin and actin) [5].

It is thus no wonder that the clinical presentation can range from apical hypertrophy, mid-ventricular hypertrophy, concentric hypertrophy and subaortic hypertrophy of the left and/or right ventricle [1,5-10].

Of all these variants, hypertrophic obstructive cardiomyopathy (HOCM) is the variant that has been studied the most [8]. In this entity (previously known as idiopathic, hypertrophic, subaortic stenosis) asymmetrical, septal hypertrophy is accompanied by the following three elements [8]: systolic anterior motion (SAM) of the anterior leaflet of the mitral valve; a left ventricular outflow tract (LVOT) gradient and mitral regurgitation.

Left ventricular false tendons are thin, fibrous or fibromuscular structures that traverse the left ventricular cavity and they may be single or multiple [11]. Recently, it was demonstrated that such a left ventricular false tendon, attached to the subaortic portion of the interventricular septum, led to striking ST-segment elevation on the surface electrocardiogram [12]. In this case report another possible diagnostic pitfall that can arise due to the presence of a subaortic tendon is presented.

## Case report

A case report is presented where it is shown that a left ventricular false tendon, when attached to the subaortic portion of the interventricular septum – a subaortic tendon – can mimic the echocardiographic appearance of hypertrophic cardiomyopathy.

A 30-year old Caucasian male was referred for a cardiovascular examination by his primary care physician. The clinical reason for the referral was the presence of a soft, subaortic midsystolic murmur (Levine grade I). The patient was totally asymptomatic and no previous medical or surgical problems were present. Clinical examination confirmed the grade I, subaortic, midsystolic murmur. The rest of the clinical examination was normal.

Echocardiography demonstrated a classical parasternal long axis picture of hypertrophic cardiomyopathy, with the phenotype of subaortic hypertrophy (see additional file 1, 2, 3 and 4). However, no systolic anterior motion of the mitral valve (SAM) and no left ventricular outflow tract gradient were present.

However, further examination revealed the presence of a big, muscular, subaortic tendon, running parallel to the interventricular septum and giving the false impression of hypertrophic cardiomyopathy. See additional file 2 demonstrating this muscular tendon clearly as a separate structure.

## Discussion

The echocardiographic assessment of ventricular hypertrophy is an extremely important component of the cardiovascular examination and it is also one of the most difficult clinical scenario's because of the vast array of pathologies, each one with a different prognosis.

Shapiro *et al *[13] performed a prospective, echocardiographic examination to determine the prevalence of localized, subaortic hypertrophy in 1000 consecutive patients presenting for a routine echocardiographic examination. They excluded patients with hypertrophic cardiomyopathy and 8 cases of such localized, subaortic hypertrophy were found. In their series, localized subaortic hypertrophy was diagnosed when the subaortic septum was 50% thicker than the mid-point of the septum.

Numerous diseases can lead to secondary left ventricular hypertrophy, which may then imitate hypertrophic cardiomyopathy [14] Jategaonkar *et al *described a case of HCM, with all the components of HOCM – asymmetric septal hypertrophy, SAM and mitral regurgitation – which turned out to be all due to an underlying pheochromocytoma [14]. Another important condition to exclude when localized, subaortic hypertrophy is found is hyperparathyroidism, as it has been shown that this condition is another important mimic of hypertrophic cardiomyopathy [15]. In the analysis of so-called "hypertrophic cardiac syndromes", they are often distinguished from one another by features such as: valvular abnormalities, outflow tract obstruction, electrocardiographic patterns, the presence or absence of diastolic dysfunction, as well as the degree and pattern of ventricular hypertrophy [16].

Amyloidosis causes the accumulation of amyloid in the myocardial interstitium and this process ultimately leads to a ventricle with a firm, rubbery consistency and ventricular hypertrophy [16]. Two-dimensional strain is a unique imaging mode that permits the objective analysis of myocardial motion throughout the entire cardiac cycle [16]. Sun *et al *[16] studied the ability of two-dimensional strain to assess global and regional function in patients with amyloidosis, hypertrophic cardiomyopathy and hypertrophy due to hypertension. They were able to demonstrate that patients with "amyloid cardiomyopathy" had significantly lower myocardial deformation as seen by two-dimensional strain imaging than patients with hypertrophic cardiomyopathy and hypertensive hypertrophy. Thus, two-dimensional strain imaging can be added to the armamentarium of the echocardiographer in the assessment of idiopathic ventricular hypertrophy.

Another good example of how a meticulous echocardiographic examination can detect the presence of a specific and unusual cause for severe ventricular hypertrophy, is Fabry's disease – an X-linked metabolic storage disease where glycosphingolipid accumulates in the myocardium and other tissues, due to deficient activity of the enzyme alpha-galactosidase A [17]. The endocardium in Fabry's cardiomyopathy has a peculiar binary appearance, detectable by transthoracic echocardiography [17].

Thus, it is clear that not all cases of subaortic hypertrophy are due to hypertrophic cardiomyopathy.

This case report adds another mimic of hypertrophic cardiomyopathy to the list – that of the muscular subaortic tendon.

## Authors' contributions

James Ker is the sole author

## Competing interests

The author declares that they have no competing interests.

## Supplementary Material

Additional file 1 **Parasternal long axis view**. This is a movie clip, demonstrating the classical echocardiographic picture of the subaortic hypertrophy variant of hypertrophic cardiomyopathy (HCM). Click here for file

Additional file 2 **Closer view of subaortic tendon**. This is a closer view of the basal interventricular septum. The thick, muscular subaortic tendon is clearly visible as a separate structure, giving the initial impression of hypertrophic cardiomyopathy. Click here for file

Additional file 3 **Parasternal long axis view**. Another movie clip, once again from the parasternal long axis view, demonstrating the subaortic hypertrophy. Click here for file

Additional file 4 **Subaortic hypertrophy**. Note the appearance of severe, subaortic hypertrophy. Click here for file
