# Supplementary figures and images for: The subaortic tendon as a mimic of hypertrophic cardiomyopathy
Source: Cardiovasc Ultrasound. 2009 Jul 3;7:31. doi: 10.1186/1476-7120-7-31 (PMC2714075; doi:10.1186/1476-7120-7-31)

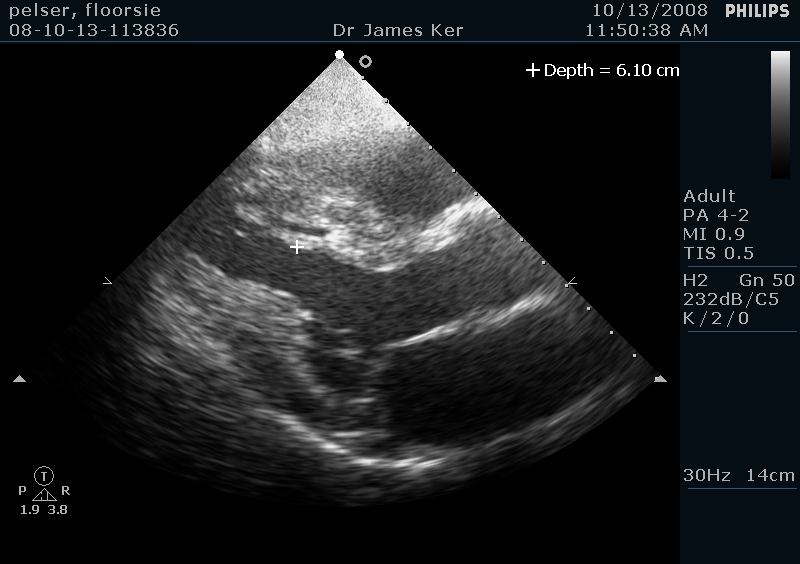

Supplement: Additional file 2 — Closer view of subaortic tendon. This is a closer view of the basal interventricular septum. The thick, muscular subaortic tendon is clearly visible as a separate structure, giving the initial impression of hypertrophic cardiomyopathy. [file 1476-7120-7-31-S2.bmp]

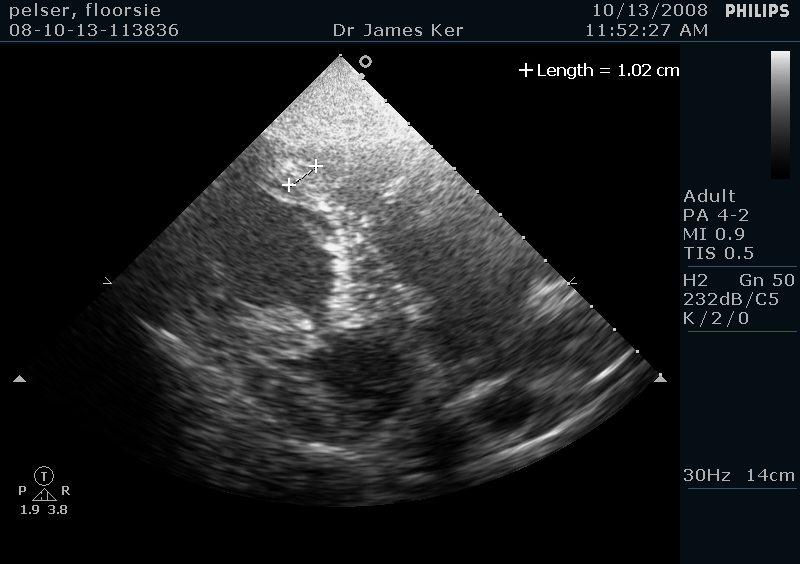

Supplement: Additional file 4 — Subaortic hypertrophy. Note the appearance of severe, subaortic hypertrophy. [file 1476-7120-7-31-S4.bmp]
